# Gender inequality among medical, pharmaceutical and dental practitioners in French hospitals: Where have we been and where are we now?

**DOI:** 10.1371/journal.pone.0254311

**Published:** 2021-07-09

**Authors:** Antoine Le Boedec, Norah Anthony, Cécile Vigneau, Benoit Hue, Fabrice Laine, Bruno Laviolle, Martine Bonnaure-Mallet, Astrid Bacle, Jean-Sébastien Allain

**Affiliations:** 1 Department of Ophthalmology, Rennes University Hospital, Rennes, France; 2 Rennes 1 University, France; 3 Methodological Support and Biostatistics Unit, University Hospital, Saint Denis, Reunion Island, France; 4 National Institute of Health and Medical Research (INSERM), Clinical Investigation Center 1410 Saint Pierre, Reunion Island, France; 5 Department of Nephrology, Rennes University Hospital, Rennes, France; 6 National Institute of Health and Medical Research (INSERM), IRSET-UMR_S 1085, France; 7 Department of Pharmacy, Rennes University Hospital, Rennes, France; 8 Clinical Investigation Unit, Rennes University Hospital, Rennes, France; 9 Department of Hepatology, Rennes University Hospital, Rennes, France; 10 National Institute of Health and Medical Research (INSERM), Clinical Investigation Center1414, France; 11 Department of Dental Surgery, Rennes University Hospital, Rennes, France; 12 National Institute of Health and Medical Research (INSERM), U1241 NuMeCAn, France; 13 Internal Medicine, Cardiovascular and Metabolism Division, Saint Malo Hospital, Saint Malo, France; Universitat de Valencia, SPAIN

## Abstract

**Introduction:**

Women are under-represented in senior academic and hospital positions in many countries. The authors aim to assess the place and the evolution of all appointed female and male health practitioners’ working in French public Hospitals.

**Materials and methods:**

Data of this observational study were collected from the National Management Centre (Centre National de Gestion) from 2015 up to January 1, 2020. First, the authors described demographic characteristics and specialties of all appointed medicine, pharmacy, and dentistry doctors’ working as Hospital Practitioners, Associate Professors, and Full Professors in French General and University-affiliated Hospitals in 2020. Then, they retrospectively reported the annual incidence of new entrance according to gender and professional status from 1999 to 2019 thanks to the appointment date of all practitioners in activity between 2015 and 2020.

**Results:**

In 2020, 51 401 appointed practitioners (49.7% of female) were in activity in French public hospitals with a large majority being medical doctors (92.4%) compared to pharmacists (6%) and dentists (1.6%). Women represented 52.5% of the Hospital Practitioners, 48.6% of the Associate Professors, and 22.0% of the Full Professors (p < 0.001). There were disparities between the rates of female Full Professors in medicine (20.6%), pharmacy (36.1%), and dentistry (44.3%, p < 0.001). Women were appointed Hospital Practitioners and Associate Professors earlier than men (respectively 37.1 versus 38.8 years, p < 0.001 and 36.1 versus 36.5 years, p = 0.04), and at a later age among Full Professors (43.7 versus 41.9 years, p < 0.001). Compared to men, the annual proportion of appointed women varied significantly between 1999 and 2019 from 47.6% to 60.4% for Hospital Practitioners, from 50.0% to 44.6% for Associate Professors, and from 11.2% to 33.3% for Full Professors (p < 0.001 for trend).

**Conclusions:**

Although more and more women occupy positions in French hospitals, there is still a gender gap regarding access to Full Professor status in medicine and pharmacy, but not in dentistry. The disparity in numbers makes comparison difficult. Despite a trend towards gender equality during the last twenty years, it has not yet been achieved regarding access to the highest positions.

## Introduction

Gender inequality is a universal issue that has recently and increasingly been addressed in public debate. These inequalities affect the functioning of society and may concern various societal areas such as fundamental rights, access to education, wage differences, and access to high professional positions [1–4]. In this context of increased awareness of gender inequalities, the under-representation of women in senior academic positions has been pointed out in the literature [5–10]. In the medical field, which is perceived as an elitist professional sector, a demographic transition "in favour" of women is globally happening [11]. However, these worldwide progressist trends should not mask the fact that male doctors still massively dominate leadership roles [12–14]. Despite more women working in the health system, female health practitioners still hit the glass ceiling regarding access to upper-level management positions. Similar findings are observed in pharmacy and dentistry [15,16].

In France, qualification as a medical doctor, pharmacist and dentist requires a specific doctorate. Apart from the first common year, training is separate for each discipline. The growing proportion of women in medical practice began in the seventies thanks to the increasing rate of women attending university [17]. In 2019, 64% of medical, pharmaceutical, and dental students were women [18]. French medical practitioners, pharmacists, and dentists can work in the private or in the public sector. The French public healthcare system includes General Hospitals and University-affiliated Hospitals. Doctors of medicine, pharmacy, and dental surgery working in public hospitals have the “Hospital Practitioner” status. This status is obtained after completion of the National public health practitioner competition. Hospital Practitioners can both work in General and University-affiliated Hospitals. They are only employed by the Hospital. For those who additionally want to pursue a university career, University-affiliated Hospitals, created in 1958, gave them access to two tenured academic positions: “Associate Professor–Hospital Practitioner” and “Full Professor–Hospital Practitioner” respectively referred to as Associate Professors and Full Professors for the rest of the article [19]. Associate and Full Professors have a triple mission of teaching, care, and research. Both Hospital and University employ them. Some can firstly become Associate Professors then Full Professors. However, many of them can be appointed Full professors at the outset. To achieve this highest academic position, Full Professors must justify many abilities like accreditation to supervise research (habilitation), overseas elective period, and publication of numerous international scientific articles in medical journals. Funders play no role in appointing Professors. The dean of their University and the National Universities Council (Conseil National des Universités, CNU) must approve their tenure. Dean is the traditional name given to the director of the Training and Research Units (Unités de Formation et de Recherche–UFR) in Universities. There is one dean for each UFR of Medicine, Pharmacy or Dentistry. The CNU is a French advisory and decision-making institution in charge of the careers of Full and Associate professors.

Although the general proportion of women in medical practice is already well documented, to this date there are few data regarding female representation and evolution of gender equality over time in medicine, pharmacy, and dentistry in France. Such granularity would be a valuable contribution to describing potential persisting areas of disparity between women and men. The primary objective of the study was to describe medicine, pharmacy, and dentistry practitioners’ gender representation in French General Hospitals and University-affiliated Hospitals as Hospital Practitioners, Associate Professors, and Full Professors in 2020. The secondary objectives were to report gender distribution according to both medical speciality and academic status in 2020 and describe the incidence of hospital and academic status appointments according to gender over the last twenty years, from 1999 to 2019.

## Materials and methods

This is an observational retrospective study based on data collected up to January 1, 2020.

### Data source

We obtained exhaustive anonymised data from the charts of personnel statistics (French Hospital Practitioners, Associate Professors, and Full Professors) from the National Management Centre (Centre National de Gestion: CNG). The CNG was created in 2007 and is in charge of the management of all appointed French doctors of medicine, pharmacy, and dental surgery working in the public healthcare sector. It is a public institution which carries out administrative duties under the authority of the French Ministry of Health. The database provided the history of each of the Hospital Practitioners, Associate Professors, and Full Professors in activity in General or University-affiliated Hospitals between 2015 and January 1, 2020. Basic sociodemographic data, gender, appointment date (or tenure), administrative status, type of workplace, discipline and medical specialty were all documented. Three main disciplines were categorized: medicine, pharmacy, and dentistry. Dentistry included oral surgery. Data concerning Associate and Full Professors in pharmacy began in 2006, the year of the legislative decree connecting their university and hospital careers [20]. In France, biology as a specialty can be practiced through both medical and pharmaceutical training. Data did not allow for the differentiation between medical doctors and pharmacists specialized in biology. We decided to categorise this speciality within the medical disciplines. On top of the medical specialties, Associate, and Full Professors have a particular university specialty under the aegis of the CNU. Specialities are slightly different between hospital practitioners and professors and do not allow for direct comparisons.

### Statistical methods

First, we described the population of practitioners’ working in French public Hospitals according to gender (especially women representation) and academic status, as of January 1, 2020. Second, we reported gender distribution according to both medical specialty and academic status in 2020. Then, we retrospectively described the gender distribution from 1999 to 2019 based on the appointment date (or tenure) of each practitioner. Descriptive and bivariate analyses of the data were performed using 3.4.2 R version R^®^. Quantitative variables are expressed as mean (standard deviation) and categorical variables as number (percentage). Quantitative variables are compared using the Student’s t-test. Categorical variables are compared using Chi square test. Evolution of the rate of female appointments over time was investigated using a Chi squared test for trend. For all analyses, a p-value < 0.05 was considered significant. Missing data were not imputed.

## Results

**Fig 1:**
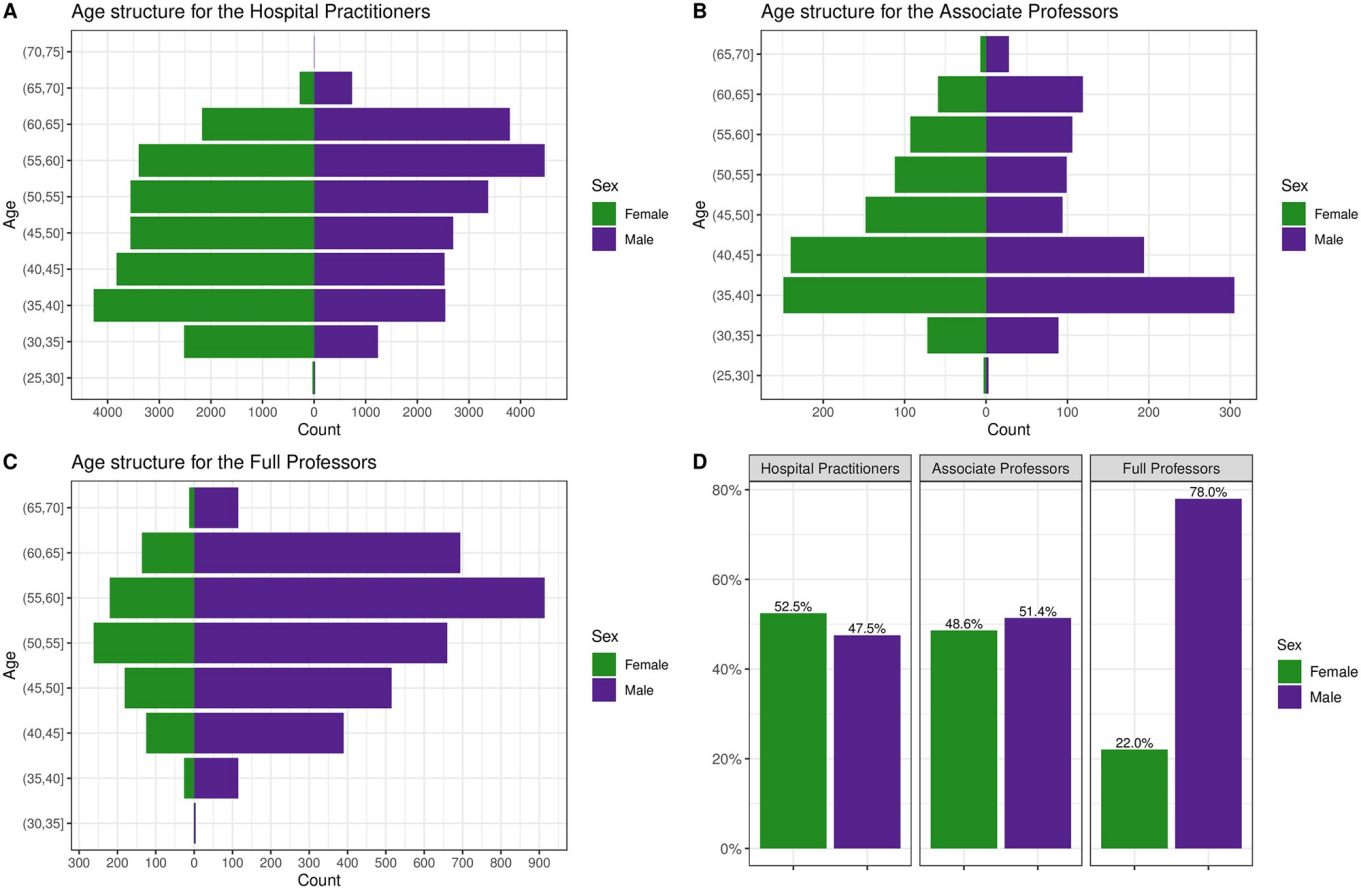
Age structure of Hospital Practitioners (A), Associate Professors (B), Full Professors (C), and gender distribution of all (D) in 2020.

As of January 1, 2020, 25 560 (49.7%) female and 25 841 (50.3%) male Health Practitioners were active in French public hospitals. A large majority of these are medical doctors (92.4%) compared to pharmacists (6.0%) and dentists (1.6%). There were 45 007 (87.6%) Hospital Practitioners, 2 022 (3.9%) Associate Professors, and 4 372 (8.5%) Full Professors. Among all of them, 20755 (40.4%) were working in University-affiliated Hospitals. Demographic characteristics of French public hospital doctors in 2020 are detailed in Table 1. All disciplines together, women represent 52.5% of the Hospital Practitioners, 48.6% of the Associate Professors, and only 22.0% of the Full Professors (p < 0.001). We represent gender and age distribution according to these statuses in Fig 1. In medicine, women represent 51.2% of Hospital Practitioners, 49.7% of Associate Professors, and 20.6% of Full Professors (p < 0.001). In pharmacy, women represent 73.5% of Hospital Practitioners, 58.0% of Associate Professors, and 36.1% of Full Professors (p < 0.001). These percentages are respectively for female dentists of 38.9%, 40.0%, and 44.3% (p = 0.49). Regarding the age of appointments, women were appointed Hospital Practitioners or Associate Professor earlier than men and at a later age among Full Professors (Table 1).

**Table 1:** Demographic characteristics of French public hospital doctors in 2020.

	Female	Male	Total	
**Hospital Practitioners, n (%)**	23613 (52.5%)	21394 (47.5%)	45007	
Age, years (SD)	47.6 (9.4)	51.6 (9.6)	49.5 (9.7)	p<0.001
Age of appointment, years (SD)	37.1 (5.8)	38.8 (6.5)	37.9 (6.2)	p<0.001
Discipline, n (%)				P<0.001
Medicine	21493 (51.2%)	20495 (48.8%)	41988	
Pharmacy	2010 (73.5%)	726 (26.5%)	2736	
Dentistry	110 (38.9%)	173 (61.1%)	283	
**Associate professors, n (%)**	983 (48.6%)	1039 (51.4%)	2022	
Age, years (SD)	45.1 (8.5)	46.0 (10.0)	45.5 (9.3)	p = 0.003
Age of appointment, years (SD)	36.1 (3.9)	36.5 (4.0)	36.3 (4.0)	p = 0.04
Discipline, n (%)				P<0.001
Medicine	735 (49.7%)	744 (50.3%)	1479	
Pharmacy	102 (58.3%)	73 (41.7%)	175	
Dentistry	146 (39.7%)	222 (60.3%)	368	
**Full professors, n (%)**	964 (22%)	3408 (78%)	4372	
Age, years (SD)	52.6 (6.8)	54.0 (7.4)	53.7 (7.3)	p<0.001
Age of appointment, years (SD)	43.7 (5.0)	41.9 (4.6)	42.3 (4.8)	p<0.001
Discipline, n (%)				P<0.001
Medicine	833 (20.6%)	3210 (79.4%)	4043	
Pharmacy	65 (36.1%)	115 (63.9%)	180	
Dentistry	66 (44.3%)	83 (55.7%)	149	

Abbreviation: SD = standard deviation.

Tables 2 and 3 describe gender distribution for each medical specialty, according to the professional status in 2020. Among hospital practitioners, there are large disparities in the proportion of male and female depending on the specialty. For example, there are 19% of female in neurosurgery, while 59% of the psychiatrist are female. Among full professor, there is always a majority of male except for the Pathology specialty (56.3% of female). Urology is the only specialty with no female among the 69 French Full Professors.

**Table 2:** Distribution of French medical doctors working as Hospital Practitioners according to specialty and gender in 2020.

	Female	Male	Total
Anaesthesiology	1724 (46.8%)	1962 (53.2%)	3686
Biology	1392 (67.6%)	668 (32.4%)	2060
Cardiovascular medicine and vascular medicine	403 (29.6%)	957 (70.4%)	1360
Clinical pharmacology	60 (71.4%)	24 (28.6%)	84
Dermatology and venereology	181 (70.2%)	77 (29.8%)	258
Digestive surgery	125 (19.2%)	525 (80.8%)	650
Emergency medicine	1893 (39.5%)	2902 (60.5%)	4795
Endocrinology, diabetology, and nutrition	450 (81.8%)	100 (18.2%)	550
Forensic medicine	52 (45.2%)	63 (54.8%)	115
Functional explorations	24 (53.3%)	21 (46.7%)	45
Gastroenterology and hepatology	414 (44.2%)	522 (55.8%)	936
General medicine	2057 (57.1%)	1543 (42.9%)	3600
General surgery	78 (24.5%)	240 (75.5%)	318
Geriatric	1588 (66.1%)	815 (33.9%)	2403
Gynaecology and obstetrics	825 (50.2%)	818 (49.8%)	1643
Haemobiology and transfusion	74 (69.2%)	33 (30.8%)	107
Haematology	209 (59.9%)	140 (40.1%)	349
Hospital hygiene	159 (74.0%)	56 (26.0%)	215
Infectious and tropical diseases	167 (56.6%)	128 (43.4%)	295
Intensive care medicine	153 (26.7%)	421 (73.3%)	574
Internal medicine and clinical immunology	336 (53.4%)	293 (46.6%)	629
Maxillofacial surgery	41 (45.6%)	49 (54.4%)	90
Medical genetics	71 (77.2%)	21 (22.8%)	92
Medical gynaecology	58 (93.5%)	4 (6.5%)	62
Nephrology	271 (49.8%)	273 (50.2%)	544
Neurology	531 (55.6%)	424 (44.4%)	955
Neurosurgery	30 (19.0%)	128 (81.0%)	158
Nuclear medicine	97 (48.7%)	102 (51.3%)	199
Occupational medicine	58 (66.7%)	29 (33.3%)	87
Oncology	173 (61.1%)	110 (38.9%)	283
Oncology-radiotherapy	63 (47.7%)	69 (52.3%)	132
Ophthalmology	185 (48.3%)	198 (51.7%)	383
Orthopaedic surgery	77 (8.3%)	848 (91.7%)	925
Otorhinolaryngology	182 (34.7%)	342 (65.3%)	524
Paediatrics	1856 (68.9%)	838 (31.1%)	2694
Paediatric surgery	96 (49.2%)	99 (50.8%)	195
Pathology	256 (70.3%)	108 (29.7%)	364
Physical medicine and rehabilitation	255 (62.0%)	156 (38.0%)	411
Plastic surgery	31 (34.8%)	58 (65.2%)	89
Pneumology	435 (50.6%)	424 (49.4%)	859
Psychiatry	3134 (59.0%)	2181 (41.0%)	5315
Public health	250 (54.9%)	205 (45.1%)	455
Radiology	680 (47.2%)	762 (52.8%)	1442
Rheumatology	208 (57.8%)	152 (42.2%)	360
Thoracic surgery	31 (15.7%)	167 (84.3%)	198
Urology	30 (8.4%)	326 (91.6%)	356
Vascular surgery	30 (20.8%)	114 (79.2%)	144

**Table 3:** Distribution of French Associate and Full Professors according to discipline, CNU-section, and gender in 2020.

	Associate Professors			Full Professors		
	Female	Male	Total	Female	Male	Total
Anatomy	9 (36.0%)	16 (64.0%)	25	6 (9.4%)	58 (90.6%)	64
Anaesthesiology	2 (8.7%)	21 (91.3%)	23	13 (10.7%)	109 (89.3%)	122
Bacteriology	76 (58.0%)	55 (42.0%)	131	35 (35.0%)	65 (65.0%)	100
Biochemistry	67 (60.4%)	44 (39.6%)	111	18 (24.3%)	56 (75.7%)	74
Biostatistics	12 (35.3%)	22 (64.7%)	34	13 (26.5%)	36 (73.5%)	49
Cardiology	6 (35.3%)	11 (64.7%)	17	10 (7.2%)	128 (92.8%)	138
Cellular biology	23 (51.1%)	22 (48.9%)	45	17 (37.8%)	28 (62.2%)	45
Child psychiatry	4 (44.4%)	5 (55.6%)	9	13 (36.1%)	23 (63.9%)	36
Dermatology	8 (66.7%)	4 (33.3%)	12	25 (37.9%)	41 (62.1%)	66
Digestive surgery	10 (40.0%)	15 (60.0%)	25	12 (9.7%)	112 (90.3%)	124
Emergency medicine	1 (12.5%)	7 (87.5%)	8	2 (5.0%)	38 (95.0%)	40
Endocrinology	10 (58.8%)	7 (41.2%)	17	21 (30.9%)	47 (69.1%)	68
Epidemiology	18 (48.6%)	19 (51.4%)	37	27 (38.6%)	43 (61.4%)	70
Forensic medicine	7 (35.0%)	13 (65.0%)	20	11 (34.4%)	21 (65.6%)	32
Gastroenterology and hepatology	5 (35.7%)	9 (64.3%)	14	14 (10.9%)	115 (89.1%)	129
General surgery	0 (0%)	0 (0%)	0	0 (0.0%)	3 (100.0%)	3
Genetics	34 (66.7%)	17 (33.3%)	51	26 (38.2%)	42 (61.8%)	68
Gynaecology and obstetrics	4 (33.3%)	8 (66.7%)	12	17 (13.5%)	109 (86.5%)	126
Haematology and transfusion	38 (50.7%)	37 (49.3%)	75	34 (26.6%)	94 (73.4%)	128
Histology	36 (70.6%)	15 (29.4%)	51	12 (25.5%)	35 (74.5%)	47
Immunology	36 (60.0%)	24 (40.0%)	60	23 (28.4%)	58 (71.6%)	81
Infectious disease	7 (41.2%)	10 (58.8%)	17	19 (25.3%)	56 (74.7%)	75
Intensive care medicine	1 (8.3%)	11 (91.7%)	12	8 (8.8%)	83 (91.2%)	91
Internal medicine and geriatrics	11 (44.0%)	14 (56.0%)	25	33 (21.6%)	120 (78.4%)	153
Maxillofacial surgery	1 (11.1%)	8 (88.9%)	9	5 (21.7%)	18 (78.3%)	23
Medical gynaecology	15 (55.6%)	12 (44.4%)	27	16 (43.2%)	21 (56.8%)	37
Nephrology	5 (45.5%)	6 (54.5%)	11	19 (24.1%)	60 (75.9%)	79
Neurology	12 (54.5%)	10 (45.5%)	22	22 (18.0%)	100 (82.0%)	122
Neurosurgery	2 (18.2%)	9 (81.8%)	11	4 (6.5%)	58 (93.5%)	62
Nuclear medicine	17 (31.5%)	37 (68.5%)	54	14 (20.6%)	54 (79.4%)	68
Nutrition	17 (81.0%)	4 (19.0%)	21	10 (25.0%)	30 (75.0%)	40
Occupational medicine	11 (55.0%)	9 (45.0%)	20	9 (37.5%)	15 (62.5%)	24
Oncology and radiotherapy	11 (42.3%)	15 (57.7%)	26	22 (17.3%)	105 (82.7%)	127
Ophthalmology	4 (44.4%)	5 (55.6%)	9	14 (21.5%)	51 (78.5%)	65
Orthopaedic surgery	2 (22.2%)	7 (77.8%)	9	3 (3.4%)	84 (96.6%)	87
Otorhinolaryngology	1 (10.0%)	9 (90.0%)	10	10 (13.0%)	67 (87.0%)	77
Parasitology	32 (61.5%)	20 (38.5%)	52	12 (38.7%)	19 (61.3%)	31
Pathology	40 (69.0%)	18 (31.0%)	58	49 (56.3%)	38 (43.7%)	87
Paediatric surgery	9 (60.0%)	6 (40.0%)	15	10 (16.7%)	50 (83.3%)	60
Paediatrics	25 (64.1%)	14 (35.9%)	39	47 (27.0%)	127 (73.0%)	174
Pharmacology	24 (43.6%)	31 (56.4%)	55	14 (23.7%)	45 (76.3%)	59
Physical medicine and rehabilitation	6 (66.7%)	3 (33.3%)	9	8 (16.7%)	40 (83.3%)	48
Physiology	36 (41.4%)	51 (58.6%)	87	26 (25.7%)	75 (74.3%)	101
Plastic surgery	3 (33.3%)	6 (66.7%)	9	5 (15.6%)	27 (84.4%)	32
Pneumology	7 (46.7%)	8 (53.3%)	15	12 (14.1%)	73 (85.9%)	85
Psychiatry	5 (23.8%)	16 (76.2%)	21	20 (22.0%)	71 (78.0%)	91
Radiology	6 (37.5%)	10 (62.5%)	16	34 (19.2%)	143 (80.8%)	177
Rheumatology	5 (55.6%)	4 (44.4%)	9	18 (23.4%)	59 (76.6%)	77
Therapeutics	6 (46.2%)	7 (53.8%)	13	8 (15.1%)	45 (84.9%)	53
Thoracic surgery	3 (37.5%)	5 (62.5%)	8	4 (4.5%)	85 (95.5%)	89
Urology	2 (33.3%)	4 (66.7%)	6	0 (0.0%)	69 (100.0%)	69
Vascular surgery and medicine	3 (42.9%)	4 (57.1%)	7	9 (12.9%)	61 (87.1%)	70

Abbreviation: CNU = National Universities Council (Conseil National des Universités).

**Fig 2:**
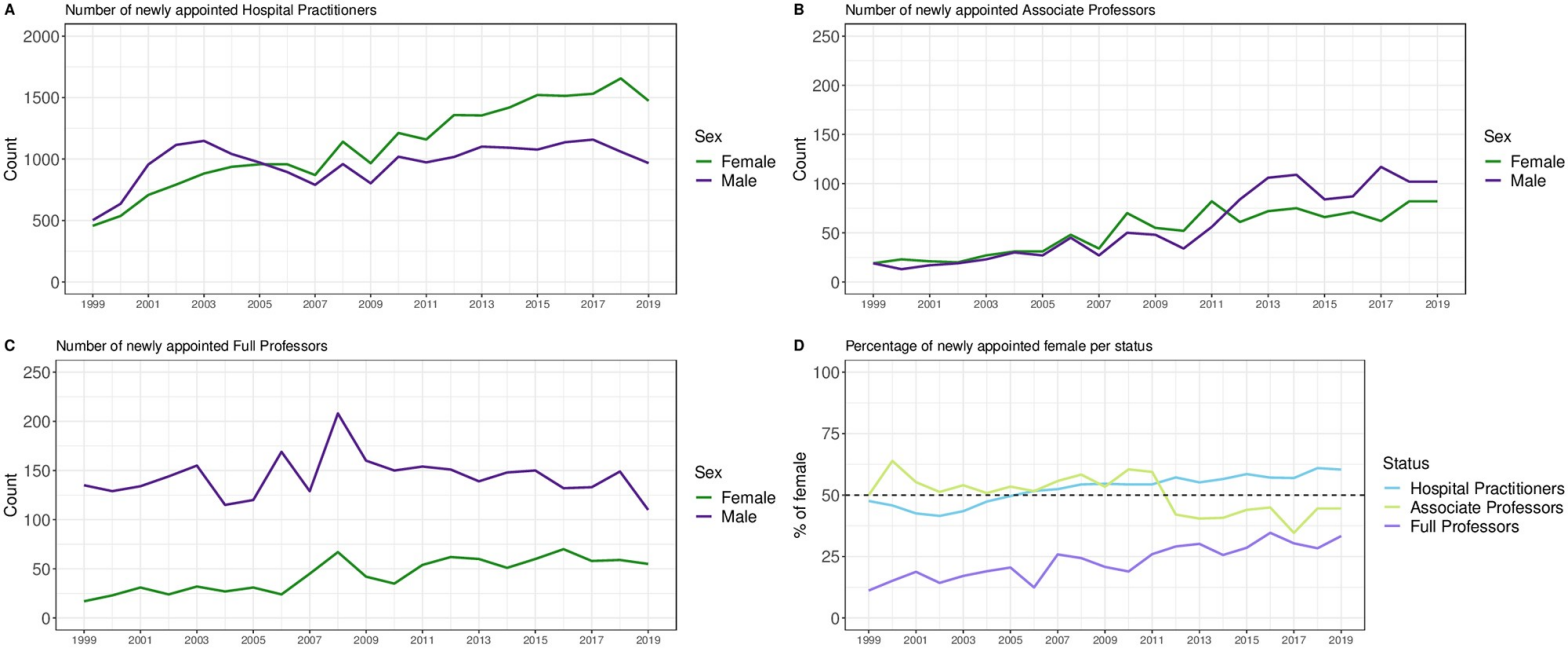
Newly appointed Hospital Practitioners (A), Associate Professors (B), Full Professors (C), and proportion of female according to the three statuses (D) between 1999 and 2019.

As shown in Fig 2, the number of newly appointed female doctors increased between 1999 and 2019, from 457 (47.6% compared to male) to 1474 (60.4%) per year for Hospital Practitioners (p < 0.001 for trend), and from 17 (11.2%) to 55 (33.3%) per year for Full Professors (p < 0.001). Even if the absolute number of female Associate Professors increase during the same period, the relative proportion decreased compare to male from 50.0% to 44.6% per year (p < 0.001 for trend). Since 2005, there have been more female appointed as Hospital Practitioners annually than male. This reversal takes place in 2012 for Associate Professors. By contrast, the number of female appointed Full Professor has been consistently lower than male appointments every year since 1999. A figure showing the evolution of newly appointed full professors according to gender and discipline between 1999 and 2019 is available in a supplemental appendix (S1 Fig).

## Discussion

Data from this study show that the representation of women in the medical field in French public hospitals varies according to academic status. We show that the gender distribution is rather balanced for Hospital Practitioners (52.5% of female) and Associate Professors (48.6%). On the other hand, less than one Full Professor in four is female in France in 2020. In addition, the percentage of female French deans is 16.2% in medicine, 16.7% in pharmacy and 43.7% in dentistry in 2020 [21–23]. This reflects a glass ceiling which excludes women only from the highest academic levels and leadership roles [24,25]. The gender distribution evolution among newly appointed Full Professors between 1999 and 2019 does not indicate enough improvement of this tendency.

Although there is clear evidence of progress, the rate of advancement of women into leadership positions in academic medicine is slower than what would be predicted by their numbers for the past twenty years in France. Several explanations could be advanced to explain this gender inequality in the Full Professor career. Many authors have already identified obstacles to the promotion of women, mainly in English or American publications. There are well-documented gender gaps in publications [26–28], peer review processes [29], grant supports [30–35], recognition awards [36–39], speaker invitations [39–42], composition of editorial boards [43–46], and leadership positions [47–50].

We also found that French women are generally appointed Full Professor at a later age than men (43.7 versus 41.9 years old). This differs from the appointment of Hospital Practitioners and Associate Professor. One could argue that the overseas elective period is mandatory to become Full Professor and may be conflicting with maternity. Medical and research training frequently coincide with childbearing and early child-rearing years.

Generally speaking, a loss of wage income is seen for French women who have children compared to women not having children. In contrast, the arrival of a child has almost no impact on men, except for men in higher paid positions who increase their activity [51].

We also cannot rule out a form of discrimination. Strong evidence suggests that the gender disparity could be due to gender differences in promotional rates [52]. In addition, tacit biases have been shown to favour men over women in science and leadership, and may affect the promotion of women in academia [52–56]. Familial factors are also obviously worth noting since women tend to be the main caregiver and time investment required for promotion, tenure, and acquisition of leadership roles is not necessarily compatible with domestic and family life. Cultural factors such as a lack of women role models, a negative gender climate sustained by a male dominated institutional environment might also be part of the equation. Indeed, mentorship is associated with increased career satisfaction, productivity, and promotion of medical faculty [13,57]. Basically, gender disparity has many roots and is most likely multifactorial. In a recent review, Edmunds, *et al*. [58] found that there was supportive evidence for many of these points: some women might be more interested in teaching than in research, women lack adequate mentors and role models, and women experience gender discrimination and bias. Conversely, evidence was discordant for other reasons such as the concern of work-life balance [58]. Some practical solutions may be proposed to reach a better and equitable state: mentoring programs, conferences for only female practitioners, conferences about gender equity, establishment of quotas in publications, and of course denunciation in journals with high impact factors [5,13,59–61]. If the establishment of fixed quotas in committees and boards is not an end in itself, it can be a good start.

One more interesting finding also emerges from our study. We note equivalent rates of female Dentists among Hospital Practitioners (38.9%), Associate Professors (40.0%), and Full Professors (44.3%) compared to medical doctors and pharmacists in 2020. Parity in dentistry is long standing, stable over twenty years but has never been promoted by a voluntarist policy. Comparison with medicine and pharmacy is not simple for many reasons. First, the number of dentists working in Public Hospitals is very low (1.6%) compared to medical doctors (92.4%) and pharmacists (6%). In addition, the dual "hospital" and "university" affiliation was not created at the same time in medicine (1958), dentistry (1990), and pharmacy (2006). In the same way, there are great disparities between disciplines and specialties. For example, surgery is largely male while pharmacy and pathology are predominantly female. These differences imply a cautious interpretation and do not allow direct comparisons. Comparison with other academic fields is also difficult because of large disparities in the proportion of female professors in France: engineering science (19%), physics (23%), chemistry (38%), law and political science (45%), language and literature (63%) [62]. Our results are therefore limited in scope and cannot be generalised to all academic fields. However, our results are consistent with worldwide studies [10,15,61,63–66]. Gender gap in high academic positions is concerning.

If the data from our study could be considered qualitatively accurate, it is not excluded that the incidence data might be slightly underestimated or even biased due to the retrospective nature of the study. Indeed, physicians appointed between 1999 and 2014 may have ceased all hospital activity before 2015 and are therefore not accounted for in our data. The proportion of these cases, which are likely to be marginal, might differ by gender and bias the incidence differences in one direction or another. Our data do not contain any other characteristics that might play a positive or negative role in academic promotion such as ethnicity, number of children, religion, political opinion or sexuality. Further research is needed to obtain a better overview of French discriminations, not limited to gender. However, based on exhaustive national data, this work is a high-quality contribution to highlight gender disparity in the medical academic world. We hope that a better overview of these realities can help to create a favourable environment to support women’s participation to take on academic and hospital leadership positions in medicine, pharmacy, and dentistry. Women in leadership positions are key to achieving gender parity in the academic field. Diversifying all levels of academic Medicine, Pharmacy, and Dentistry in terms of gender is a way to make our institutions better by enabling varied perspectives to be shared. Change will not happen without decision-makers’ involvement. Gender parity should be treated as a priority and sufficient resources should be allocated to make it happen.

## Supporting information

S1 Fig Newly appointed Full Professors in medicine (A), in pharmacy (B), in dentistry (C), and proportion of female Full Professors in the three disciplines (D) between 1999 and 2019. (PDF)
