# Supplementary figures and images for: Gender inequality among medical, pharmaceutical and dental practitioners in French hospitals: Where have we been and where are we now?
Source: PLoS One. 2021 Jul 9;16(7):e0254311. doi: 10.1371/journal.pone.0254311 (PMC8270123; doi:10.1371/journal.pone.0254311)

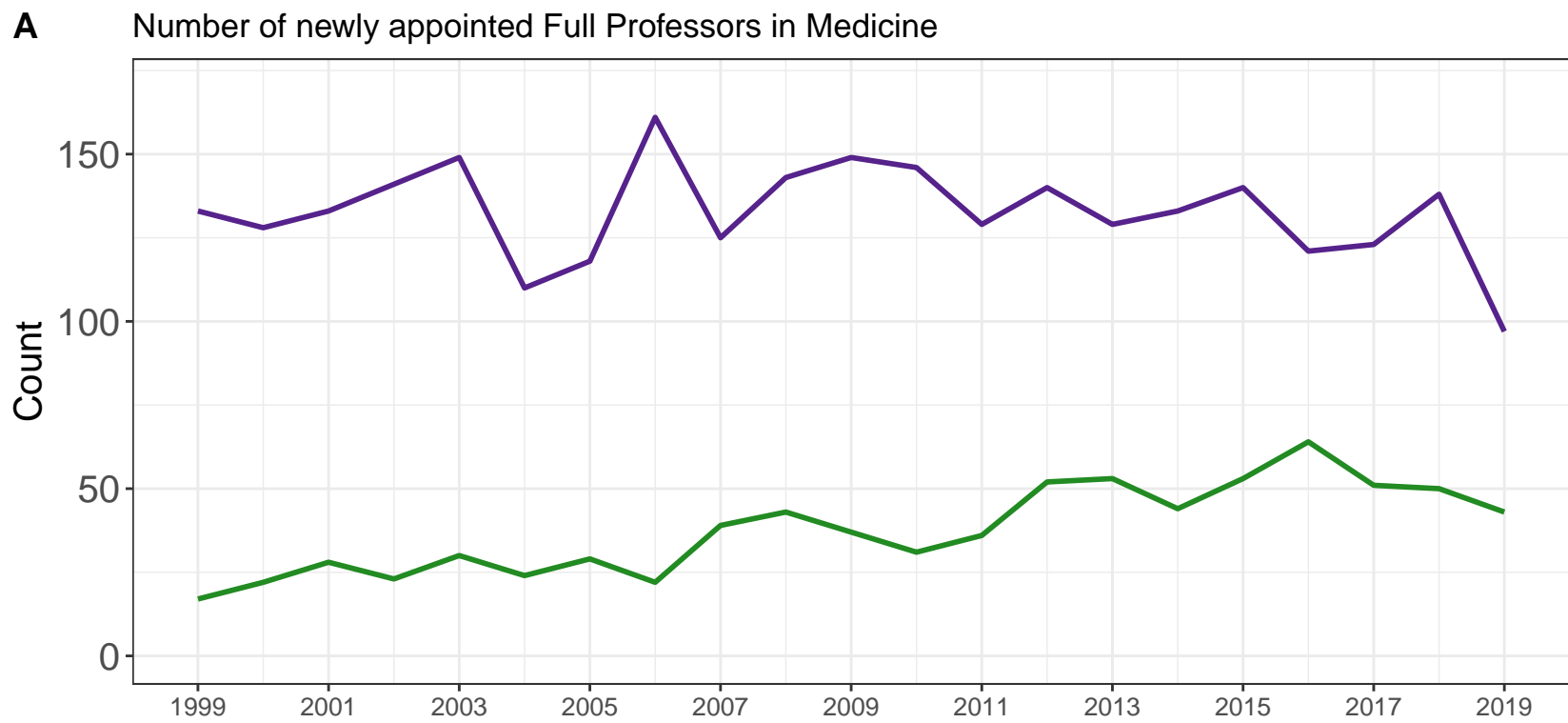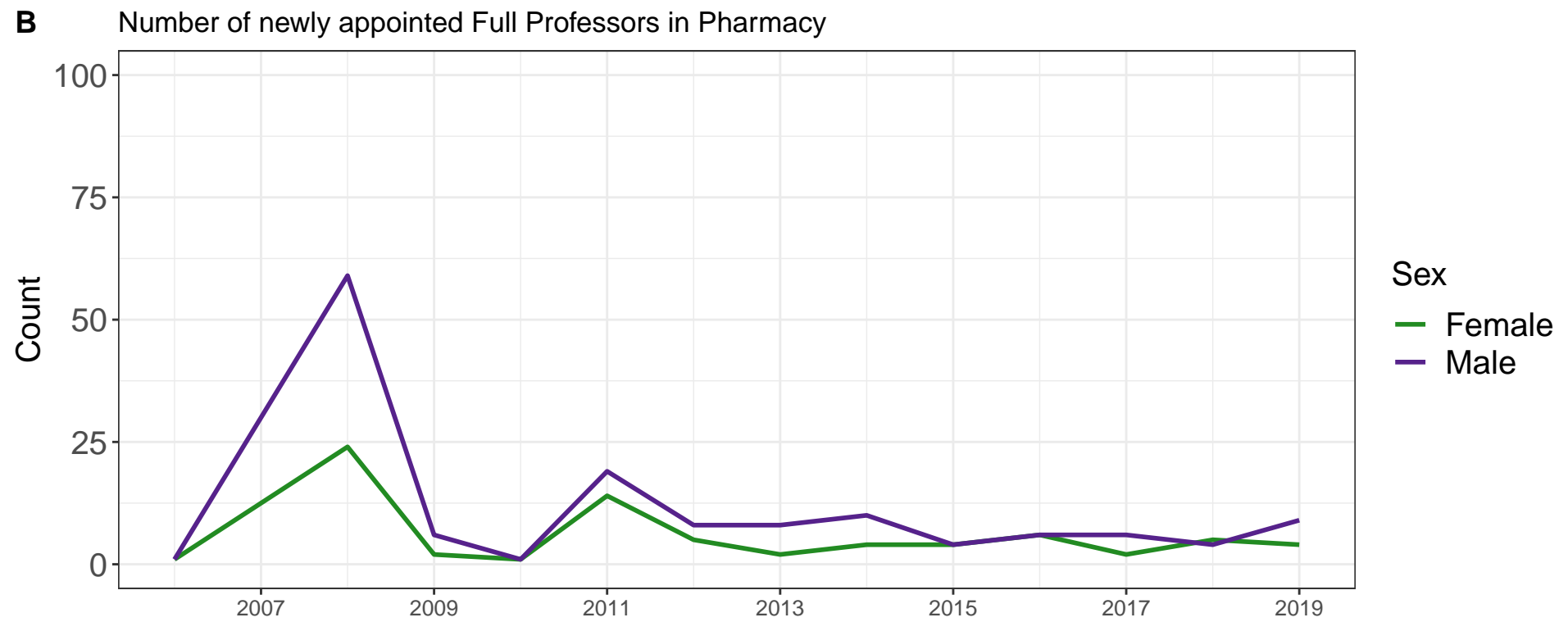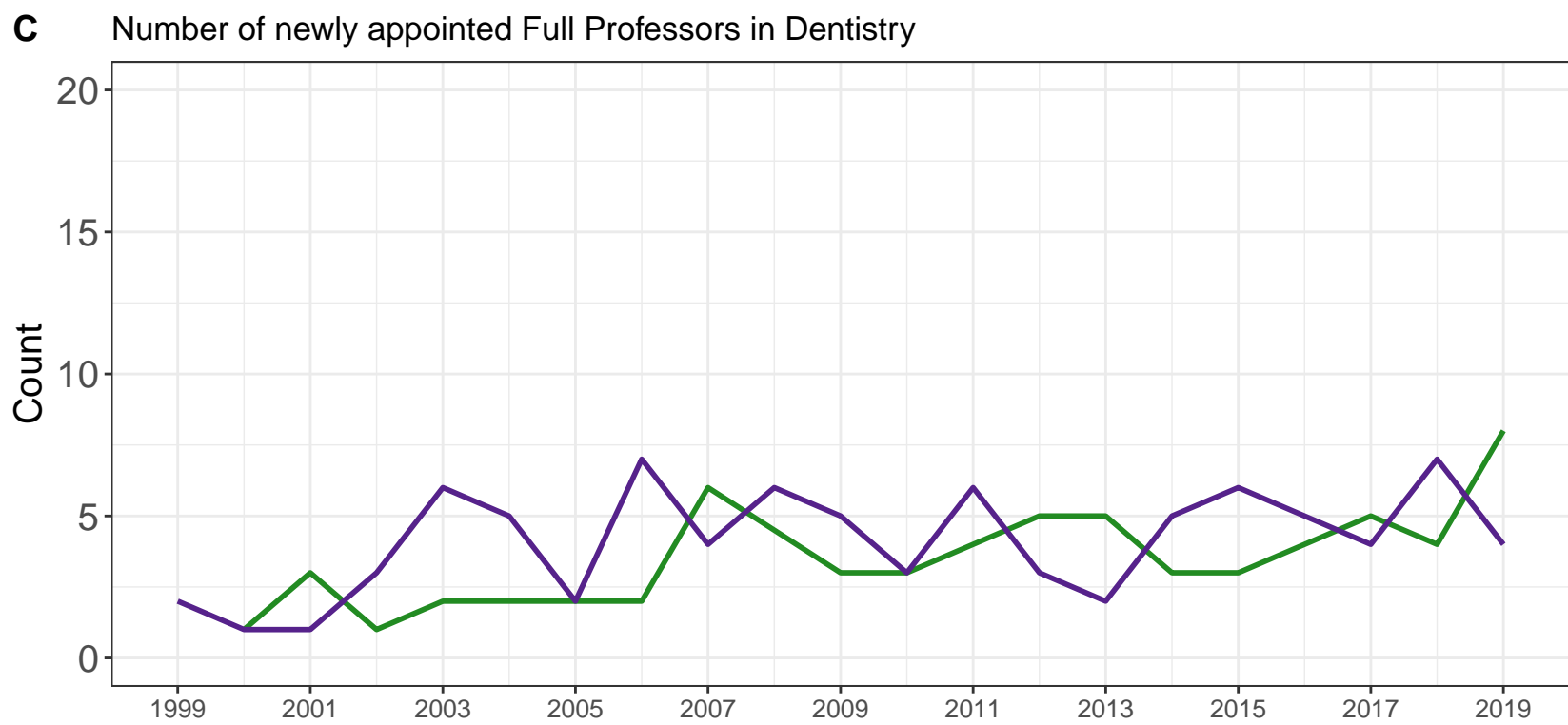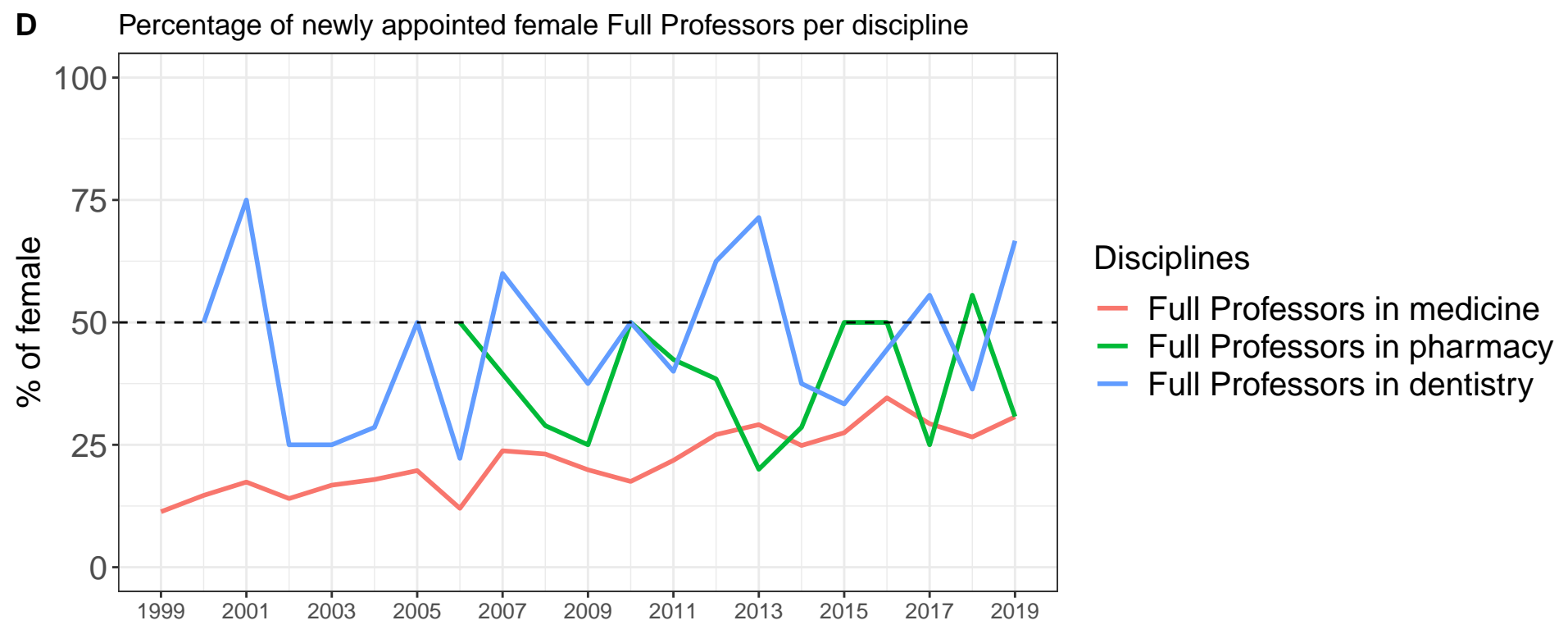

Supplement: S1 Fig — (PDF) [file pone.0254311.s001.pdf]
